# Policy and advanced monitoring synergies to tackle ammonia emission impacts

**DOI:** 10.1016/j.eehl.2026.100248

**Published:** 2026-05-20

**Authors:** Yajie Shu, Tian Hu, Ming Zhou

**Affiliations:** a National Local Joint Laboratory for Advanced Textile Processing and Clean Production, Wuhan Textile University, Wuhan 430200, China; b ENVISION Unit, Luxembourg Institute of Science and Technology, Belvaux, Sanem 4422, Luxembourg; c School of Environment and Science, Gold Coast Campus, Griffith University, Gold Coast, QLD 4222, Australia; d Australian Rivers Institute, Griffith University, Brisbane, QLD 4111, Australia

**Ammonia is a critical yet under-regulated driver of global PM_2.5_ pollution and ecosystem degradation. This commentary proposes a synergistic framework to bridge the governance gap by integrating advanced monitoring technologies, from satellite retrievals to ground-based sensors, with cross-sectoral policies and economic instruments. This integrated approach enables precise, cost-effective emission control and paves the way for a nitrogen circular economy, offering co-benefits for air quality, climate, and public health.**

Ammonia (NH_3_), the primary form of reactive nitrogen and dominant alkaline gas in the atmosphere, exerts widespread impacts on environmental quality, including air pollution, soil acidification, and water eutrophication [1]. In the atmosphere, NH_3_ acts as a key precursor of secondary particulate matter (PM_2.5_), contributing an estimated 32% (25%–39%) of the N-share during haze events in 2013 [2]. It also exacerbates air pollution via aerosol chemistry like HONO formation, indirectly raising respiratory/cardiovascular risks. Moreover, high ammonia exposure directly triggers systemic inflammation and organ damage through oxidative stress [3]. Beyond health, NH_3_ emissions drive substantial socio-economic and ecological consequences, including 23.3 million years of life lost globally and widespread nitrogen deposition that accelerates eutrophication and ecosystem degradation [2,4].

Yet, despite its wide-ranging impacts, NH_3_ has received far less policy attention than SO_2_ and NO_2_. This commentary addresses this gap by advocating for integrated policy frameworks, supported by advanced monitoring, to position NH_3_ within multi-pollutant strategies. We examine global emission trends and policy limitations and propose a synergistic framework that links precision monitoring with cross-sectoral governance. By leveraging innovations such as AI-driven satellite retrievals and real-time ground monitoring, this approach bridges data gaps, improves regulatory alignment, and enables region-specific controls. Ultimately, it supports a nitrogen circular economy while protecting ecosystems and human health amid changing environmental conditions.

## 1 Global NH_3_ emissions and policy gaps

Global anthropogenic NH_3_ emissions reached approximately 50.6 Tg N/a in 2023, with agriculture (fertilizer and livestock) accounting for 60%–80% of the total emissions [5]. However, non-agricultural sources are becoming increasingly important in regions such as East Asia, North America, and Europe. These sources include industrial NH_3_ slip from flue gas denitrification, urban activities, and combustion-related releases. Satellite observations reveal that regional disparities in emissions are particularly pronounced. For example, China’s NH_3_ emissions are estimated to be 62% higher than inventory-based values, especially in intensive agricultural regions [6]. These uncertainties limit the effectiveness of mitigation strategies and highlight the need for spatially resolved source-specific monitoring and integrated governance approaches.

For decades, NH_3_ emissions remained largely overlooked in environmental governance, despite global atmospheric levels doubling since the 1960s due to agricultural intensification. Early actions in Europe and North America, such as the Gothenburg Protocol (1999) and the EU’s National Emission Ceilings Directive (2001/81/EC), recognized NH_3_ as a transboundary pollutant, but achieved smaller reductions than for SO_2_ and NO_x_ [2]. In North America, where non-agricultural sources are significant, the regulation is less centralized. The U.S. regulates NH_3_ mainly under the Clean Air Act through State Implementation Plans targeting PM_2.5_, with a focus on animal feeding emissions. Asia, where more than half of global emissions now occur, began systematic control only in the past decade. China’s Air Pollution Prevention and Control Action Plan (2013) and the subsequent Blue Sky Protection Campaign marked the beginning of policy efforts, achieving significant PM_2.5_ reductions by targeting agricultural emissions. Recently, both the EU and China have been integrating ammonia control into broader green and low-carbon strategies through the revised Renewable Energy Directive and Five-Year Plan [7].

Yet, despite such advances, efforts remain fragmented and disproportionately focused on agriculture, leaving industrial and urban contributions unaddressed. The variation in policy focus, from international protocols in Europe to domestic air quality and carbon neutrality goals in China, highlights a persistent gap in coherent, multi-sectoral governance. Bridging these gaps requires integrated, cross-sector frameworks linking monitoring, regulation, and innovation.

## 2 A synergistic NH_3_ mitigation framework

**Fig. 1:**
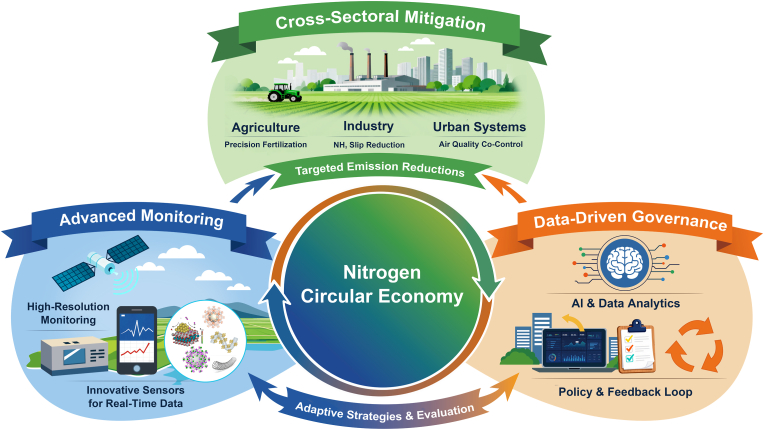
A synergistic framework linking advanced NH_3_ monitoring, cross-sectoral mitigation, and data-driven governance.

The persistent gap between NH_3’_s environmental importance and its regulatory neglect stems largely from a mismatch between data and policy. Emissions are highly variable across space and time, yet governance relies on static inventories that systematically underestimate true levels. Addressing this gap requires a paradigm shift: moving from fragmented, sector-specific measures toward a holistic strategy that integrates advanced monitoring, policy coordination, economic incentives, and technological transformation. Several synergistic frameworks to guide this transition are proposed (Fig. 1).

### 2.1 Intelligent monitoring and technological innovation

Intelligent monitoring and technological innovation are transforming ammonia governance by shifting from data scarcity to precise perception. A persistent challenge in ammonia regulation lies in the lack of reliable detection under real-world conditions, where trace concentrations, high humidity, and interfering gases undermine data accuracy and limit regulatory enforceability. Recent advances in sensing materials and device engineering have been directed toward overcoming these constraints, enabling sensitive, selective, and stable detection across complex environments. Breakthroughs in functional materials [8,9], including noble-metal-doped polymers, low-dimensional nanocomposites, and heterostructure design, have enabled next-generation sensors with ppb-level sensitivity, improved humidity tolerance, and reduced cross-interference. These developments extend monitoring capabilities beyond laboratory settings and support deployment in real-world regulatory contexts [10].

More importantly, these technologies are translated into actionable governance tools. For example, innovations in scalable fabrication (e.g., liquid metal printing [11], inkjet printing [12]) enable low-cost, flexible sensors suitable for decentralized deployment in wearable devices and food packaging. Meanwhile, the convergence of self-powering mechanisms (e.g., triboelectric nanogenerators) with Internet of Things (IoT) architectures facilitates distributed monitoring networks capable of long-term, unattended operation. These systems generate continuous, high spatiotemporal-resolution data on ammonia concentrations and fluxes across atmospheric, soil, and aquatic environments. Such data streams support adaptive governance by enabling dynamic adjustment of regulatory thresholds, targeted interventions, and improved nitrogen management. Ultimately, the transition from isolated sensing devices to interconnected monitoring ecosystems marks a shift toward evidence-based, data-driven, and responsive ammonia governance.

### 2.2 Cross-sectoral mitigation strategies

Ammonia governance has traditionally focused on agriculture, while industrial, traffic, and urban sources remain under-regulated. A more effective strategy requires cross-sectoral integration. In agriculture, real-time monitoring, informed by wind-controlled enhancement systems, should guide spatiotemporally optimized fertilizer application, with measured reductions tied to subsidies or ecological compensation. For instance, improving nitrogen use efficiency via inhibitors or deep placement can reduce NH_3_ emissions by 75% and CO_2_eq by 20% [13]. In industry, stricter control of NH_3_ slip in flue-gas treatment is essential, and catalytic technologies can achieve high N_2_ selectivity and low NO_x_ emissions.

Cross-sectoral coordination also enables synergies with NO_x_ and SO_2_ reduction, which together maximize PM_2.5_ abatement efficiency. Indeed, evidence [2] shows that NH_3_ abatement is up to ten times more cost-effective than that of NO_x_, and co-reducing NH_3_ and NO_x_ delivers the greatest overall benefit. To harness these efficiencies, economic tools such as integrating NH_3_ into emissions trading or creating a dedicated nitrogen market would align agricultural and industrial incentives. Such tools can address emissions shifts associated with international trade, transforming NH_3_ mitigation from a sectoral obligation into a systemic governance strategy.

### 2.3 Data-driven governance systems

Advanced monitoring achieves policy impact only when embedded in governance systems that transform data into regulatory decisions. Establishing a monitor–evaluate–adjust feedback loop is therefore critical, in which monitoring data directly supports compliance verification, hotspot identification, and adaptive policy adjustments. At the macro scale, satellite-based monitoring enables top-down detection of emission patterns and prioritization of hotspots. AI-enhanced satellite retrievals, such as IASI NH_4_ columns (capable of identifying CAFO-related hotspots at 1 km resolution) [14], and global NH_3_ maps from instruments like HIRAS [15] provide systematic, large-scale identification of major emission areas. These tools enable regulators to verify regional compliance with emission ceilings, prioritize inspections, and guide the targeted deployment of ground-level mitigation measures. By combining satellite data with ground-based measurements, a more accurate picture of NH_3_ emissions emerges, ensuring interventions are both spatially precise and effective.

At the regional scale, digital twin platforms integrate multi-source data, including meteorology, facility-level emissions, and trade flows, to simulate policy scenarios and inform decision-making. Models that quantify canopy-layer NH_3_ deposition or track carbon-nitrogen flows via life cycle assessment [13] help assess mitigation potentials and predict shifts in NH_3_ emissions. By linking real-time monitoring data with scenario analysis, these platforms support adaptive policy adjustments, allowing regulators to refine emission standards, optimize nitrogen management strategies, and respond to emerging pollution patterns.

In sum, integrated dynamic mapping and digital simulation platforms close the policy-data loop. By converting high-resolution monitoring data into actionable regulatory insights, these systems enable more transparent, targeted, and responsive ammonia governance.

## 3 Outlook: A nitrogen circular economy

Achieving sustained NH_3_ mitigation requires structural transformation that repositions NH_3_ from a pollutant to a resource within a practical nitrogen circular economy framework. This transition depends on integrating technological innovation, economic instruments, and data-driven governance. Scaling up green ammonia production using renewable hydrogen, combined with carbon capture, utilization, and storage, can reduce both carbon and nitrogen footprints. Coupling this with nutrient recovery from waste streams helps close the nitrogen loop. Economic instruments, including nitrogen pricing and emissions trading, can incentivize circular practices, supported by regulatory targets for nitrogen use efficiency and ammonia recovery across industrial and agricultural sectors. Equally important is robust data-driven governance. Intelligent monitoring networks and digital twin platforms enable real-time tracking of nitrogen flows, verification of mitigation outcomes, and adaptive optimization through continuous feedback loops. Advancing integrated monitoring, cross-sector coordination, and policy alignment will be essential to operationalize this transition. Ultimately, embedding NH_3_ management within a circular economy framework offers a pathway to cleaner air, climate co-benefits, and improved ecosystem resilience.

## CRediT authorship contribution statement

**Yajie Shu:** Conceptualization, Data curation, Formal analysis, Writing – original draft. **Tian Hu:** Formal analysis, Writing – review & editing. **Ming Zhou:** Conceptualization, Formal analysis, Supervision, Writing – review & editing.

## Declaration of competing interests

The authors declare no competing interests.

## References

[1] Erisman J.W. (2021). How ammonia feeds and pollutes the world. Science.

[2] Gu B., Zhang L., Van Dingenen R., Vieno M., Van Grinsven H.J., Zhang X. (2021). Abating ammonia is more cost-effective than nitrogen oxides for mitigating PM_2.5_ air pollution. Science.

[3] Ma R., Li K., Guo Y., Zhang B., Zhao X., Linder S. (2021). Mitigation potential of global ammonia emissions and related health impacts in the trade network. Nat. Commun..

[4] Zhang X., Gu B., van Grinsven H., Lam S.K., Liang X., Bai M. (2020). Societal benefits of halving agricultural ammonia emissions in China far exceed the abatement costs. Nat. Commun..

[5] Soulie A., Granier C., Darras S., Zilbermann N., Doumbia T., Guevara M. (2024). Global anthropogenic emissions (CAMS-GLOB-ANT) for the Copernicus Atmosphere Monitoring Service simulations of air quality forecasts and reanalyses. Earth Syst. Sci. Data.

[6] Ma J., Shi H., Zhu Y., Li R., Wang S., Lu N. (2025). The evolution of global surface ammonia concentrations during 2001–2019: magnitudes, patterns, and drivers. Environ. Sci. Technol..

[7] Xu P., Li G., Zheng Y., Fung J.C.H., Chen A., Zeng Z. (2024). Fertilizer management for global ammonia emission reduction. Nature.

[8] Lv W., Yang J., Xu Q., Mehrez J.A., Shi J., Quan W. (2024). Wide-range and high-accuracy wireless sensor with self-humidity compensation for real-time ammonia monitoring. Nat. Commun..

[9] Sun X., Chen T., Liang Y., Zhang C., Zhai S., Sun J. (2023). Enhanced sensitivity of SAW based ammonia sensor employing GO-SnO_2_ nanocomposites. Sens. Actuators, B Chem..

[10] Gasso S., Carrier J., Radu D., Lai C.-Y. (2024). Novel gas sensing approach: ReS_2_/Ti_3_C_2_T_*x*_ heterostructures for NH_3_ detection in humid environments. ACS Sens..

[11] Nguyen C.K., Taylor P.D., Zavabeti A., Alluhaybi H., Almalki S., Guo X. (2023). Instant-in-air liquid metal printed ultrathin tin oxide for high-performance ammonia sensors. Adv. Funct. Mater..

[12] Liu C., Bai Y., Li W., Yang F., Zhang G., Pang H. (2022). *In situ* growth of three-dimensional MXene/metal-organic framework composites for high-performance supercapacitors. Angew. Chem. Int. Ed..

[13] Luo L., Wang K., Liu S., Liu H., Tong L., He L. (2024). Tracking carbon and ammonia emission flows of China’s nitrogen fertilizer system: implications for domestic and international trade. Environ. Sci. Technol..

[14] Epps A., Dressel I.M., Guo X., Odanibe M., Fields K.P., Carlton A.M.G. (2025). Satellite observations of atmospheric ammonia inequalities associated with industrialized swine facilities in eastern north *Carolina*. Environ. Sci. Technol..

[15] Zhou M., Deng Z., Robert C., Zhang X., Zhang L., Wang Y. (2024). The first global map of atmospheric ammonia (NH_3_) as observed by the HIRAS/FY-3D satellite. Adv. Atmos. Sci..

